# Fatigue Is Associated With Diminished Cardiovascular Response to Anticipatory Stress in Patients With Coronary Artery Disease

**DOI:** 10.3389/fphys.2021.692098

**Published:** 2021-08-17

**Authors:** Julija Gecaite-Stonciene, Brian M. Hughes, Julius Burkauskas, Adomas Bunevicius, Nijole Kazukauskiene, Lisanne van Houtum, Julija Brozaitiene, Julius Neverauskas, Narseta Mickuviene

**Affiliations:** ^1^Laboratory of Behavioral Medicine, Neuroscience Institute, Lithuanian University of Health Sciences, Palanga, Lithuania; ^2^School of Psychology, National University of Ireland, Galway, Ireland; ^3^Department of Clinical Psychology, Leiden University, Leiden, Netherlands

**Keywords:** stress, cardiovascular response, fatigue, physiology, coronary artery disease, acute coronary syndrome, cardiovascular measures, cardiovascular dysfunction

## Abstract

**Background:**

Fatigue and psychophysiological reactions to mental stress are known to be problematic in coronary artery disease (CAD) patients. Currently, studies exploring the relationship between fatigue and cardiovascular reactivity to stress are scarce and inconsistent. The current study aimed to investigate the links between cardiovascular response to mental stress and fatigue in CAD patients after acute coronary syndrome (ACS).

**Methods:**

The cross-sectional study investigated 142 CAD patients (85% males, 52 ± 8 years) within 2–3 weeks after recent myocardial infarction or unstable angina pectoris. Fatigue symptoms were measured using Multidimensional Fatigue Inventory 20-items, while cardiovascular reactivity to stress [i.e., systolic (S) and diastolic (D) blood pressure (ΔBP), and heart rate (ΔHR)] was evaluated during Trier Social Stress Test (TSST). In addition, participants completed psychometric measures, including the Hospital Anxiety and Depression scale and the Type D Scale-14. Multivariable linear regression analyses were completed to evaluate associations between fatigue and cardiovascular response to TSST, while controlling for confounders.

**Results:**

After controlling for baseline levels of cardiovascular measures, age, gender, education, heart failure severity, arterial hypertension, smoking history, use of nitrates, anxiety and depressive symptoms, Type D Personality, perceived task difficulty, and perceived task efforts, cardiovascular reactivity to anticipatory stress was inversely associated with both global fatigue (ΔHR: β = –0.238; *p* = 0.04) and mental fatigue (ΔSBP: β = –0.244; *p* = 0.04; ΔHR β = –0.303; *p* = 0.01) as well as total fatigue (ΔSBP: β = –0.331; *p* = 0.01; ΔHR: β = –0.324; *p* = 0.01).

**Conclusion:**

In CAD patients after ACS, fatigue was linked with diminished cardiovascular function during anticipation of a mental stress challenge, even after inclusion of possible confounders. Further similar studies exploring other psychophysiological stress responses are warranted.

## Introduction

Despite medical advances that have improved coronary artery disease (CAD) outcomes over the past fifty years, CAD remains the most common cause of premature mortality, accounting for almost one third of all deaths worldwide (Piepoli et al., 2016; Al-Mallah et al., 2018). CAD typically includes diagnoses of angina pectoris, and may lead to acute coronary syndrome (ACS) (De Lemos and Omland, 2017). Acute stress can act as a trigger for ACS, such as myocardial infarction and unstable angina pectoris (Amsterdam et al., 2014). Meanwhile, prolonged or chronic stress is considered an important risk factor for the development and progression of cardiovascular diseases (Holmes et al., 2006).

The traditional reactivity hypothesis states that higher cardiovascular response to emotional stressors contributes to the etiology of cardiovascular pathology (Steptoe and Kivimäki, 2012). However, a growing body of evidence posits that maladaptive regulation of the autonomic nervous system (ANS) can be expressed not only by exaggerated but also by blunted cardiovascular response to stress, both of which may signal physiological dysregulations (Lovallo, 2011). Prolonged psychological stress may disrupt biological regulatory systems, such as ANS, and so may lead to maladaptive blunted cardiovascular response (Hughes et al., 2018). Low cardiovascular responses to stress have been found to be associated with indices of poor health, such as physical disability (Phillips et al., 2011) and obesity (Phillips et al., 2012). Blunted cardiovascular response have also been associated with characteristics of psychological distress in cardiac patients, including Type D (*Distressed)* personality (Denollet, 2005) as well as anxiety and depressive symptoms (Howard et al., 2011; Kupper et al., 2013; Gecaite et al., 2019). Even though there are no clear cut-off points for determining whether the cardiovascular response is exaggerated or diminished (often referred to as “blunted”) (Turner, 1994), a burgeoning scientific literature suggests that blunted stress reactions are themselves associated with adverse health outcomes (Phillips et al., 2013).

Fatigue, defined as the multidimensional subjective experience of persistent and extreme tiredness, mental and physical exhaustion (Smets et al., 1995; Ream and Richardson, 1996; Aaronson et al., 1999), is one of the most frequent and distressing symptoms reported by patients with CAD after ACS (Burkauskas et al., 2017; Johnston and Eckhardt, 2018). In patients with CAD, the prevalence of moderate to severe fatigue has been reported to be as high as 39% during cardiac rehabilitation (CR), remaining up to 28% after 1 year (van Geffen et al., 2015). Meanwhile, mental fatigue is considered a risk factor for the development of heart diseases (Guan et al., 2017), while unusual fatigue is a strong predictor of longer prehospital delay (Zègre-Hemsey et al., 2018), poor health-related outcomes, and increased mortality (Irvine et al., 1999). Considering fatigue within the context of emotional stress, it may not only be a distressing symptom itself but also may play an important role in determining the effort and motivation to perform a task (Wright, 2014), and so in turn may shape variations of cardiovascular response to stress (Wright, 2009).

To date, there have been several studies suggesting positive associations between higher fatigue and higher perceived stress in patients with CAD after ACS (Cameron et al., 2005; Fredriksson-Larsson et al., 2015). However, to our knowledge, no study has examined links between psychophysiological stress parameters, such as cardiovascular response to stress, and fatigue in patients with heart related conditions (Johnston and Eckhardt, 2018), even though the psychophysiological research underlying the interplay between ANS and fatigue in this population is often proposed (Matura et al., 2018). Only several earlier studies in different populations reported that heart rate (HR) reactivity to mental stressor was lower in patients with chronic fatigue syndrome (CFS) than in healthy controls (Soetekouw et al., 1999). Meanwhile, significant inverse associations between the systolic blood pressure (SBP) and diastolic BP (DBP) response during mental stress and fatigue severity were detected in young women (Nolte et al., 2008). Nevertheless, conflicting results (Wright et al., 2008) and non-significant associations (Nelesen et al., 2008) have also been documented.

Considering the limited and inconsistent research regarding fatigue and its effects on cardiovascular response during mental stress, the current study aimed to investigate the relationship between cardiovascular response to mental stress and subjective fatigue in patients with CAD after ACS. Taking into account the results from previous studies (Soetekouw et al., 1999; Nolte et al., 2008), it was hypothesized that higher subjective fatigue would be associated with diminished cardiovascular response (ΔSBP; ΔDBP; ΔHR) to mental stress, even after possible confounders were considered.

## Materials and Methods

### Study Procedure

A total of 251 consecutive patients with CAD attending an inpatient CR clinic were invited to participate in this study. Inclusion criteria were: (a) meeting diagnostic criteria for ACS as evaluated by a cardiologist; (b) attending a standard CR program based on previously established secondary prevention guidelines for CAD (Steg et al., 2012); (c) no history of arrhythmic disorder and/or implantation of a cardioverter defibrillator; and (d) ability to communicate in the local vernacular language (Lithuanian). Exclusion criteria were: (a) severe comorbid illness; (b) unstable cardiovascular condition as determined by a cardiologist; (c) age above 80; (d) significant communicative difficulties or cognitive impairment; and (e) unwillingness to participate in the study. In sum, 92 (36.7%) potential participants were excluded from the study. The final sample thus comprised 159 participants (84.5% men, age 53 ± 8.2 years).

After an informed consent form was signed by the patient, clinical and sociodemographic data were gathered, primarily from medical records during the first 2–3 days of admission to CR. The clinical data included formal diagnosis, severity of heart failure as classified using the New York Heart Association (NYHA) functional classification standard (Levin et al., 1994), left ventricular ejection fraction, percutaneous coronary intervention, arterial hypertension (AH), obesity [body mass index (BMI) > 30 kg/m^2^], medication use, and smoking behavior. Sociodemographic data comprised age, education, and gender. Self-report assessment for fatigue, depression and anxiety symptoms, Type D personality, and mental stress test were completed within 10 days after admission. The Trier Social Stress Test (TSST) was employed to measure cardiovascular response (ΔSBP,- ΔDBP, and ΔHR) before, during and after mental stress (Kirschbaum et al., 1993). Participants were asked to have a standard breakfast and lunch and were instructed to abstain from food 1 h prior to the afternoon session as well as from caffeine, alcohol, exercise, soda, beverages etc. The study patients were instructed to use beta-blockers in the day the experimental procedures took place, according to their treatment plan designated by the cardiologist. The experimental session of procedure took place at 2:30 p.m. The procedure room is always maintained the optimal temperature between 22 and 25°C. After the TSST, participants were debriefed about the goal of the experiment and remaining questions were answered.

This study was conducted in accordance with the principles stated in the Declaration of Helsinki and was approved by the Kaunas Regional Ethics Committee for Biomedical Research in Lithuania. All patients were monitored by the cardiologist during the study process in order to ensure their medical safety.

### Measures

#### Multidimensional Fatigue Inventory, MFI-20

Fatigue was evaluated using the original version of the Multidimensional Fatigue Inventory (MFI-20) created by Smets and colleagues (Smets et al., 1995). The MFI-20 includes five subscales: General fatigue, physical fatigue, mental fatigue, reduced activity, and reduced motivation. Each subscale has 4 items with possible answers on a 5-point Likert scale. General fatigue is assessed using general statements about overall fatigue, and reduced functioning is assessed based on questions related to how fatigue affects daily functioning. The physical fatigue subscale involves questions about physical feelings related to fatigue. Mental fatigue relates to cognitive functioning, including concentration problems. Reduced activity refers to the influence of physical and psychological factors on the level of activity. Reduced motivation relates to the lack of motivation to start any activity due to tiredness. Adequate psychometrics properties (Cronbach’s coefficients α of all scales ranged between 0.70 and 0.80) were found in previous studies using this scale with patients with CAD in Lithuania (Staniute et al., 2014). The MFI-20 has also been recently validated in a large sample of CAD patients (Gecaite-Stonciene and Bunevicius, 2020). The total score ranges from 4 to 20 on each subscale, and 20–100 for total fatigue score with a higher score indicating higher fatigue levels.

#### Hospital Anxiety and Depression Scale, HADS

The Hospital Anxiety and Depression Scale (HADS) was employed to assess patients’ anxiety and depressive symptoms during the past week (Snaith, 2003). HADS is a self-administered, 14-item questionnaire, comprised of two independent subscales for measuring anxiety (HADS-A, 7 items) and depressive (HADS-D, 7 items) symptoms. It is based on 4-point Likert scale (0–3), with total scores ranging from 0 to 21 per subscale, while a higher sum of scores representing greater symptom severity. Cut-off values of ≥ 8 were considered for screening significant anxiety (HADS-A) and depressive (HADS-D) symptoms. In a previous study with patients with CAD, HADS showed acceptable internal consistency, Cronbach‘s coefficients α = 0.84 for HADS-A, and α = 0.60 for HADS-D (Gecaite et al., 2019).

#### Type D Scale-14, DS-14

The Type D Scale-14 (DS14) (Denollet, 2005) was employed to determine the presence of Type D personality, which is a common clinical risk factor in patients with CAD (Kupper and Denollet, 2018). Overall, the DS14 is comprised of 14-items, having two subscales of negative affectivity (NA) (7 items) and social inhibition (SI) (7 items). The total score of ≥ 10 on both subscales indicates the presence of Type D personality. Previous studies in patients with CAD indicated good psychometric parameters of DS14 (Cronbach α = 0.75) (Gecaite et al., 2019).

#### Mini-Mental State Examination, MMSE

The Mini-Mental State Examination (MMSE) was employed to test for possible cognitive impairment (Folstein et al., 1975). This instrument is divided into two parts with an overall maximum score of 30. The first section covers orientation, memory and attention abilities (total score range from 0 to 21). The second section includes the evaluation of ability to name, follow verbal/written instructions, and ability to write sentences and to copy a complex polygon figure (total score range from 0 to 9) (Folstein et al., 1975). The total sum of scores of the two sections ≤ 24 is considered to represent cognitive impairment (Blake et al., 2002; Burton and Tyson, 2015). Previous studies have reported high internal consistency for the MMSE (Cronbach α = 0.91) (Marioni et al., 2011).

#### Trier Social Stress Test, TSST

Stress responses were evaluated based on a conventional Trier Social Stress Test (TSST) (Kirschbaum et al., 1993). The first phase of TSST started with 10 min of baseline rest (Kirschbaum et al., 1993). Next, participants were asked to prepare a 5-min free form speech for a simulated job interview to convince the committee members that they were the best applicant for a job. Anticipatory stress was taken to represent the period starting with the task instructions, during which patients were introduced to the evaluating committee of three trained researchers. Cardiovascular measures were recorded during speech preparation. Participants were then given time to make an interview-style presentation (5 min). Following this, participants underwent a mental arithmetic test (8 min). The arithmetic task comprised a Paced Auditory Serial Addition Test (Gronwall, 1977). After this, the TSST concluded with a 10-min recovery period, during which participants rested on their own.

Cardiovascular measures (i.e., SBP, DBP, and HR) were assessed during each stage of the TSST, and were recorded every 2 min (e.g., during task instruction at 1, 3, and 5 min) before being averaged for each stage. Detailed procedural nuances of this standardized TSST are reported elsewhere (Kirschbaum et al., 1993; Birkett, 2011; Frisch et al., 2015; Allen et al., 2017). Our procedure was based on previously established protocols (Kirschbaum et al., 1993) with modifications only to the arithmetic task, as reported elsewhere (Girdler et al., 2005; Mechlin et al., 2007; Cox et al., 2015).

Cardiovascular parameters were recorded using a Oscar2 24-HR ABP monitor (SunTech Medical Inc., Morrisville, NC, United States) (Jones et al., 2004). Participants were observed so that the TSST could be terminated if the participant were to be at risk of excessively elevated BP, defined for CAD patients as BP ≥ 210/115 mmHg (Fletcher et al., 2013).

#### Visual Analog Scales, VAS

During the study, patients filled in two Visual Analog Scales (VAS) 5 min after the TSST, in order to measure perceived efforts and perceived difficulty of the tasks. This method was chosen based on previously established study protocols involving the TSST (von Dawans et al., 2011). The two scales ranged from 0 (maximum difficulty/efforts) to 100 (minimum difficulty/efforts). The method of VAS is considered a valid and applicable tool to evaluate subjective experience in various experimental clinical settings (Wewers and Lowe, 1990).

### Statistical Analysis

Statistical analyses were completed using SPSS version 17.0. To test for assumptions, normality was assessed using the Kolmogorov-Smirnov test and skewness and kurtosis were scrutinized. Descriptive statistics were generated to summarize clinical and sociodemographic characteristics. The internal validity of all scales was calculated using α-coefficients of Standardized Cronbach.

In order to confirm that the TSST induced laboratory-based social stress in this sample, a linear mixed model was computed. Based on this analysis, the mean differences between cardiovascular parameters of baseline and other TSST stages were evaluated.

Due to relatively small number of participants, analysis for identification of outliers was completed and outliers were eliminated from the further analysis. Univariate outliers were considered if z-scores were greater than 2.26 (*p* < 0.001, two-tailed). Mahalanobis distances, using a chi-square cut-off (*p* < 0.001), were employed in order to identify and eliminate multivariate outliers.

Measures of cardiovascular response were obtained by subtracting the averaged values of the HR, SBP and DBP during baseline rest from the averaged scores during other TSST periods. Two-tailed Student’s *t*-test, Mann Whitney *U* test and Fisher’s χ^2^ were used to evaluate patients’ characteristics of socio-demographic and clinical data, fatigue, depressive/anxiety symptoms, Type D personality, as well as cardiovascular response to stress. The analysis for multicollinearity was also performed by using variance inflation factor (VIF) values < 4.

Univariate linear regression analysis was completed to measure the links between cardiovascular response to TSST and fatigue, sociodemographic/clinical characteristics, anxiety/depressive symptoms, Type D personality (in order to identify possible confounders). Benjamini-Hochberg adjustment for multiple comparisons was applied to control for Type I error, setting a critical value for false discovery rate of 0.10 (Benjamini and Hochberg, 1995). Multivariable linear regression was used to assess associations between cardiovascular response to TSST and fatigue, while controlling for possible confounders.

Covariates were chosen either based on the previous literature or those which were significant in univariate models. This procedure was followed based on earlier established statistical guidelines on multivariable linear analysis in order to test the hypothesis on associations between the variables when controlling for covariates (Katz, 2011).

## Results

Based on the criteria outlined above, 17 univariate outliers were identified, which were removed from the analysis. Thus, the remaining analysis was completed with 142 study patients. In terms of internal consistency, all study questionnaires and subscales showed adequate Cronbach α-coefficients (Tavakol and Dennick, 2011) as depicted in Table 1, and were used in the remaining analysis.

**TABLE 1:** Internal consistency results for MFI-20, HADS, and DS14 (*N* = 142).

	Mean	SD	Number of items	Standardized Cronbach’s α
**Fatigue (MFI-20 domains)**				

General fatigue	12.33	4.99	4	0.82
Physical fatigue	13.98	5.53	4	0.84
Reduced activity	15.20	4.98	4	0.83
Reduced motivation	11.37	4.19	4	0.79
Mental fatigue	11.59	4.82	4	0.81
Total fatigue score	51.58	16.09	20	0.93

**Mental distress (HADS subscales)**				

Anxiety symptoms (HADS-A)	5.22	3.47	7	0.82
Depressive symptoms (HADS-D)	3.24	2.56	7	0.72
Type D personality (DS14)	18.67	9.70	14	0.83

**MFI-20, Multidimensional Fatigue Inventory; HADS, Hospital Anxiety and Depression Scale; HADS-A, subscale for anxiety symptoms; HADS-D, subscale for depressive symptoms; DS14, The Type D Scale.**

### Sample Characteristics

As presented in Table 2, patients’ mean age was 52 years (SD = 8.2), and were predominantly males (85.2%) with mostly high school degrees (47.2%) and College/University degrees (52.8%). More than half of the participants met the criteria for obesity (51.4%) and had either past or present experience of nicotine use (58.5%). Most of the patients were admitted to the hospital due to acute myocardial infarction (73.2%), while the rest met the criteria for unstable angina pectoris (26.8%). According to the NYHA functional classification system, most patients met the criteria for Class II (82.6%), representing limitation of physical activity but comfort at rest. The majority had a comorbid diagnosis of AH (82.4%) and all were under current pharmacological treatment. 4.9% (*n* = 7) had moderate to severe systolic dysfunction (left ventricular ejection fraction < 40%), 91% (*n* = 129) had percutaneous coronary intervention. All participants were within the normal range of cognitive functioning, and thus were able to comprehend the instructions of the scales and complete the TSST. Around one third (29.6%) of the patients met the criteria for Type D personality and had significant anxiety symptoms (26.1%), while 8.5% of the patients has significant depressive symptoms. The detailed information of baseline characteristics is depicted in Table 2.

**TABLE 2:** Descriptive statistics for demographics and clinical characteristics (*n* = 142).

Age, mean ± SD (years)	52 ± 8.2
**Education, *n* (%)**	

High school	64 (47.2)
College/University degree	75 (52.8)

**Diagnosis, *n* (%)**	

Unstable angina pectoris	38 (26.8)
Acute myocardial infarction	104 (73.2)

**Medication Use, *n* (%)**	

Nitrates	16 (11.3)
Beta-blockers	122 (85.9)
Angiotensin-converting enzyme inhibitors	118 (83.1)
Diuretics	10 (7.0)
Benzodiazepines	9 (6.3)

**New York Heart Association functional class, *n* (%)**	

I	3 (16.2)
II	113 (79.6)
III	6 (4.2)
Percutaneous coronary intervention, *n* (%)	129 (90.8)
Left ventricular ejection fraction < 40%, *n* (%)	7 (4.9)
Obesity (BMI > 30 kg/m^2^)	73 (51.4)
Arterial hypertension, *n* (%)	117 (82.4)
Nicotine use (Smoking currently/in the past), *n* (%)	83 (58.5)
fGlobal cognitive function (Mini Mental State Examination), mean ± SD	28.16 ± 1.47
Presence of type D personality (DS14), *n* (%)	42 (29.6)

**Anxiety symptoms (Hospital Anxiety and Depression scale), *n* (%)**	

Total Score < 8	105 (73.9)
Total Score ≥ 8	37 (26.1)
**Depression symptoms (Hospital Anxiety and Depression scale), *n* (%)**	
Total Score < 8	130 (91.5)
Total Score ≥ 8	12 (8.5)

**Perceived difficulty/efforts of the Trier Social Stress Test tasks**	
**(Visual Analog Scale), mean ± SD**	

Difficulty	46.07 ± 30.68
Efforts	26.20 ± 23.71

**Fatigue (Multidimensional Fatigue Inventory-20) scores, mean ± SD**	

Global fatigue	12.33 ± 4.99
Physical fatigue	13.98 ± 5.53
Activity reduction	15.20 ± 4.98
Motivation reduction	11.37 ± 4.19
Mental fatigue	11.59 ± 4.82
Total fatigue Score	51.58 ± 16.09

### Baseline Characteristics of Stress Evoking Task (TSST)

**FIGURE 1:**
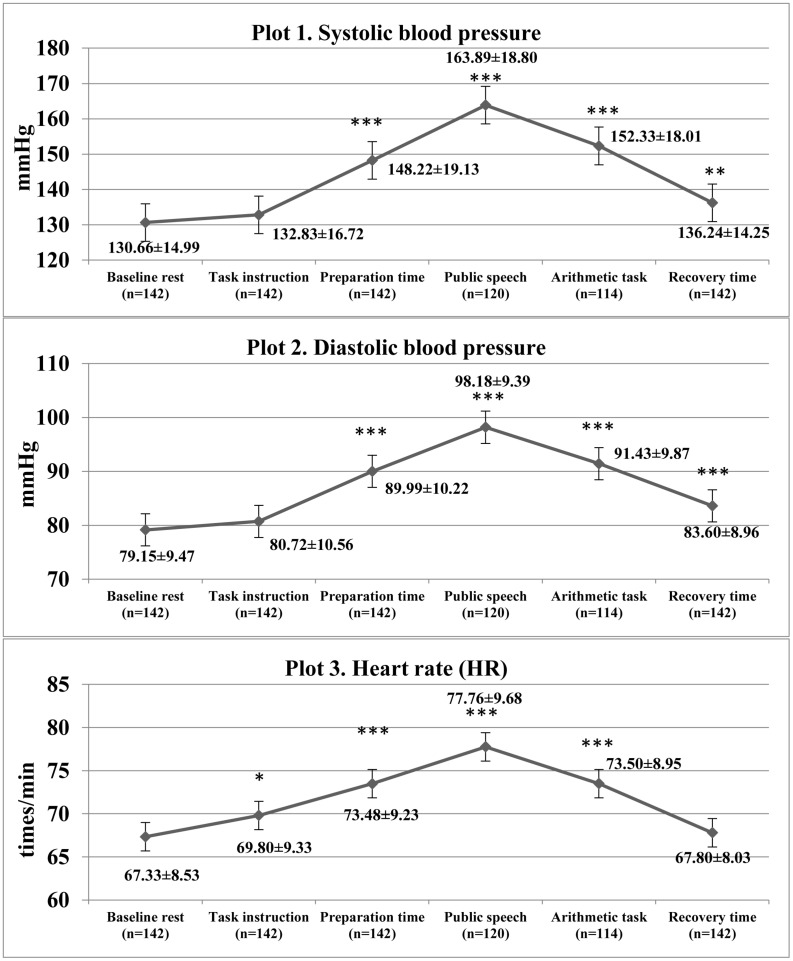
Descriptive statistics of cardiovascular characteristics and comparison with baseline cardiovascular measures during Trier Social Stress Test in the study patients. ^∗^*p* < 0.05, ^∗∗^*p* < 0.01, ^∗∗∗^*p* < 0.001 as compared with baseline rest.

The results of the linear mixed models confirmed that there was a significant increase in cardiovascular parameters as a response to TSST [SBP: *F*(5,795) = 75.38, *p* < 0.001, η^2^ = 0.322; DBP: *F*(5, 795) = 72.24, *p* < 0.001, η^2^ = 0.312]; HR: [*F*(5, 795) = 26.24, *p* < 0.001, η^2^ = 0.142; see Figure 1]. Of the participants, 22 (15.5%) did not complete the job interview task and a further 6 (4.2%) discontinued the TSST at the arithmetic task, due to maladaptive increases in cardiovascular parameters.

### Univariate Linear Regression Analysis

Univariate regression analysis showed that global fatigue was significantly linked with ΔHR during the TSST task instruction (anticipatory stress): Mental fatigue was significantly associated with ΔSBP, ΔDBP and ΔHR, while the total fatigue was significantly associated with ΔSBP and ΔHR during TSST task instruction (anticipatory stress; see Table 3). Reduction in self-reported motivation was associated with ΔSBP during TSST arithmetic task (see Table 3), however after adjustment for multiple comparisons, this association was no longer significant and so was not included in further analysis. In addition, cardiovascular responses to the TSST were significantly linked with several sociodemographic and clinical characteristics (see Table 4), which were included in the further analysis as possible covariates.

**TABLE 3:** The associations between cardiovascular reactivity during Trier Social Stress Test (TSST) in six stages and different domains of fatigue in study patients.

	Task instruction (*n* = 142)			Preparation time (*n* = 142)			Job interview (*n* = 120)			Arithmetic task (*n* = 114)			Recovery time (*n* = 142)		
	ΔHR	ΔSBP	ΔDBP	ΔHR	ΔSBP	ΔDBP	ΔHR	ΔSBP	ΔDBP	ΔHR	ΔSBP	ΔDBP	ΔHR	ΔSBP	ΔDBP
MFI-20 Global fatigue	**–0.177 (0.035)**	–0.163 (0.05)	–0.160 (0.06)	–0.081 (0.34)	–0.136 (0.11)	–	–0.077 (0.40)	–0.083 (0.37)	–0.033 (0.72)	–0076 (0.42)	–0.172 (0.07)	–0.179 (0.06)	0.052 (0.54)	–0.059 (0.48)	–0.022 (0.80)
MFI-20 Physical fatigue	–0.139 (0.10)	–0.146 (0.08)	–0.079 (0.35)	–0.039 (0.65)	–0.066 (0.44)	0.012 (0.89)	–0.036 (0.70)	–0.015 (0.88)	0.007 (0.94)	–0.068 (0.47)	–0.096 (0.31)	–0.127 (0.18)	0.022 (0.80)	0.021 (0.80)	–0.016 (0.85)
MFI-20 Activity reduction	–0.152 (0.07)	–0.132 (0.12)	–0.075 (0.38)	–0.020 (0.82)	–0.143 (0.09)	0.032 (0.71)	–0.120 (0.19)	–0.110 (0.23)	0.043 (0.64)	–0.103 (0.27)	–0.190 (0.043)	–0.144 (0.13)	0.002 (0.98)	–0.043 (0.61)	–0.041 (0.63)
MFI-20 Motivation reduction	–0.149 (0.08)	–0.134 (0.11)	–0.114 (0.18)	–0.081 (0.34)	–0.116 (0.17)	–0.107 (0.21)	–0.096 (0.30)	–0.125 (0.18)	–0.101 (0.27)	–0.097 (0.31)	–0.205 (0.029)	–0.164 (0.08)	–0.037 (0.67)	–0.073 (0.39)	–0.166 (0.048)
MFI-20 Mental fatigue	**–0.248 (0.003)**	**–0.207 (0.013)**	**–0.195 (0.020)**	–0.078 (0.36)	–0.151 (0.07)	–0.113 (0.18)	–0.030 (0.75)	–0.102 (0.27)	–0.066 (0.47)	–0.122 (0.20)	–0.137 (0.15)	–0.112 (0.24)	–0.088 (0.30)	–0.006 (0.94)	–0.116 (0.17)
MFI-20 Total fatigue score	**–0.210 (0.012)**	**–0.191 (0.023)**	–0.151 (0.07)	–	–0.147 (0.08)	–0.042 (0.62)	–0.085 (0.36)	–0.101 (0.27)	–0.032 (0.73)	–0.111 (0.24)	–0.176 (0.07)	–0.173 (0.07)	–0.009 (0.91)	–0.036 (0.67)	–0.082 (0.33)

*Univariate linear regression analyses, r’s (p).*

*SBP, systolic blood pressure; DBP, diastolic blood pressure; HR, heart rate. MFI-20, Multidimensional Fatigue Inventory 20-items.*

*To measure cardiovascular stress reactivity (delta scores), the averaged values of the HR, sBP and dBP during baseline rest were substracted from the averaged values during other TSST phases.*

*In bold significant correlations (p < 0.05).*

**TABLE 4:** The associations between cardiovascular reactivity during Trier Social Stress Test in six stages and sociodemographic and clinical factors.

	Task instruction (*n* = 142)			Preparation time (*n* = 142)			Job interview (*n* = 120)			Arithmetic task (*n* = 114)			Recovery time (*n* = 142)		
	ΔHR	ΔSBP	ΔDBP	ΔHR	ΔSBP	ΔDBP	ΔHR	ΔSBP	ΔDBP	ΔHR	ΔSBP	ΔDBP	ΔHR	ΔSBP	ΔDBP
Gender	0.056 (0.51)	0.086 (0.31)	–0.078 (0.36)	**0.191 (0.023)**	**0.199 (0.018)**	0.073 (0.39)	0.044 (0.63)	0.091 (0.32)	–0.122 (0.19)	0.008 (0.93)	0.021 (0.83)	–0.156 (0.10)	–0.081 (0.34)	–0.065 (0.45)	**–0.247 (0.003)**
Age	–0.088 (0.30)	0.027 (0.75)	–0.050 (0.56)	–0.071 (0.40)	0.090 (0.29)	–0.076 (0.37)	0.088 (0.34)	**0.211 (0.021**)	–0.082 (0.38)	0.038 (0.69)	**0.192 (0.041)**	–0.088 (0.35)	0.026 (0.76)	0.094 (0.27)	–0.104 (0.22)
BMI	–0.120 (0.15)	–0.048 (0.57)	–0.079 (0.35)	–0.129 (0.13)	0.002 (0.99)	–0.101 (0.24)	–0.024 (0.79)	–0.094 (0.31)	**–0.272 (0.003**)	–0.063 (0.51)	–0.177 (0.06)	**–0.308 (0.001)**	0.120 (0.16)	–0.163 (0.05)	–0.128 (0.13)
Education	0.148 (0.08)	0.086 (0.31)	–0.004 (0.97)	0.148 (0.08)	0.109 (0.20)	0.089 (0.29)	0.190 (0.038)	0.092 (0.32)	0.026 (0.78)	0.071 (0.45)	0.123 (0.19)	0.135 (0.15)	0.027 (0.75)	–0.006 (0.94)	0.032 (0.71)
NYHA	–0.013 (0.88)	–0.047 (0.58)	**–0.173 (0.040)**	0.027 (0.75)	0.030 (0.72)	0.043 (0.61)	0.042 (0.65)	0.060 (0.51)	0.009 (0.92)	0.038 (0.69)	0.016 (0.86)	–0.065 (0.49)	0.021 (0.80)	0.039 (0.64)	–0.033 (0.70)
Arterial hypertension	–0.060 (0.48)	–0.086 (0.31)	–0.049 (0.56)	–0.128 (0.13)	0.128 (0.13)	–0.010 (0.91)	–0.147 (0.11)	0.061 (0.51)	–0.019 (0.83)	0.005 (0.96)	0.150 (0.11)	0.145 (0.13)	–0.045 (0.60)	0.028 (0.74)	0.016 (0.85)
History of smoking	–0.008 (0.93)	**0.173 (0.039)**	0.157 (0.06)	–0.135 (0.11)	–0.007 (0.94)	0.059 (0.49)	–0.180 (0.05)	0.003 (0.98)	0.119 (0.20)	–0.166 (0.08)	–0.082 (0.39)	0.007 (0.94)	**–0.188 (0.025**)	0.069 (0.42)	0.049 (0.57)
Nitrates	0.152 (0.07)	0.076 (0.37)	**0.219 (0.009**)	0.148 (0.08)	0.131 (0.12)	0.074 (0.38)	0.093 (0.31)	0.075 (0.41)	0.035 (0.71)	0.093 (0.32)	0.031 (0.74)	–0.145 (0.12)	0.087 (0.30)	0.006 (0.94)	–0.060 (0.48)
Beta-blockers	–0.050 (0.56)	0.007 (0.93)	–0.046 (0.59)	0.031 (0.72)	0.148 (0.08)	0.133 (0.12)	–0.005 (0.96)	0.106 (0.25)	0.011 (0.91)	0.054 (0.57)	0.147 (0.12)	0.105 (0.27)	–0.022 (0.80)	0.007 (0.94)	–0.072 (0.39)
ACE inhibitors	0.087 (0.30)	0.072 (0.40)	0.060 (0.48)	–0.122 (0.15)	0.033 (0.70)	0.017 (0.84)	–0.068 (0.46)	0.055 (0.55)	0.122 (0.18)	0.104 (0.27)	0.140 (0.14)	0.176 (0.06)	0.010 (0.90)	0.039 (0.65)	0.027 (0.75)
Diuretics	–0.015 (0.86)	–0.019 (0.83)	–0.077 (0.36)	–0.105 (0.21)	**–0.313 (< 0.001**)	**–0.198 (0.019)**	–0.144 (0.12)	**–0.285 (0.002)**	–0.171 (0.06)	–0.056 (0.55)	–**0.261 (0.005**)	–**0.225 (0.016)**	0.035 (0.68)	–**0.168 (0.045)**	–0.085 (0.32)
Benzodiazepines	–0.099 (0.24)	–0.088 (0.30)	–0.070 (0.41)	0.011 (0.90)	0.022 (0.80)	–0.044 (0.61)	0.044 (0.63)	0.094 (0.31)	0.031 (0.74)	–0.016 (0.87)	0.027 (0.78)	–0.072 (0.44)	0.121 (0.15)	0.120 (0.16)	0.033 (0.70)
Anxiety symptoms (HADS-A)	–**0.208 (0.013)**	–**0.211 (0.012)**	–0.098 (0.25)	–0.005 (0.95)	0.004 (0.96)	0.048 (0.57)	–0.048 (0.60)	0.058 (0.53)	0.069 (0.46)	–0.068 (0.47)	–0.073 (0.44)	–0.069 (0.47)	–0.032 (0.71)	–0.019 (0.83)	–0.073 (0.39)
Depressive symptoms (HADS_D)	–0.085 (0.31)	–0.014 (0.87)	0.020 (0.81)	–0.154 (0.07)	**–0.200 (0.018)**	–0.160 (0.06)	–0.081 (0.38)	0.009 (0.92)	–0.039 (0.67)	–**0.081 (0.39**)	–0.136 (0.15)	–0.101 (0.29)	–0.026 (0.76)	–0.083 (0.33)	–0.132 (0.12)
Type D personality (DS-14)	–0.043 (0.61)	–0.039 (0.65)	–0.042 (0.62)	–0.058 (0.50)	–0.047 (0.58)	–0.047 (0.58)	–0.007 (0.94)	–0.005 (0.96)	–0.014 (0.88)	0.004 (0.96)	–0.058 (0.54)	–0.048 (0.61)	0.061 (0.47)	–0.159 (0.06)	–0.119 (0.16)
Perceived difficulty (VAS)	–0.102 (0.32)	0.071 (0.49)	–0.023 (0.83)	0.032 (0.75)	0.103 (0.32)	0.147 (0.15)	–0.008 (0.94)	0.145 (0.20)	0.131 (0.24)	0.036 (0.76)	0.222 (0.054)	0.140 (0.23)	–0.085 (0.41)	–0.063 (0.54)	0.004 (0.97)
Perceived efforts (VAS)	–0.089 (0.39)	0.032 (0.76)	**–0.203 (0.046)**	–0.110 (0.28)	–0.130 (0.21)	–0.182 (0.08)	–0.027 (0.81)	–0.172 (0.13)	–0.183 (0.10)	–0.001 (0.99)	–0.148 (0.20)	–0.143 (0.22)	0.075 (0.47)	0.035 (0.73)	–0.071 (0.49)

*Pearson correlation r’s (p). NYHA, New York Heart Association; HADS-A, Hospital Anxiety and Depression Scale, anxiety symptoms subscale.*

*Univariate linear regression analyses, r’s (p).*

*HADS-D, Hospital Anxiety and Depression Scale, depressive symptoms subscale; DS14, Type D Scale-14; VAS, Visual Analog Scale.*

*SBP, systolic blood pressure; DBP, diastolic blood pressure; HR, heart rate.*

*(in bold) Significant associations (p < 0.05).*

*To measure cardiovascular stress reactivity (delta scores), the averaged values of the HR, sBP, and dBP during baseline rest were substracted from the averaged values during other TSST phases.*

*In bold significant correlations (p < 0.05).*

### Multivariable Linear Regression Analysis

The main study hypotheses were tested using multivariable linear regression models. Multivariate outliers based on the analysis of Mahalanobis distance were not detected in any of the models. Analyses for multicollinearity showed adequate results (VIF values < 4). The covariates included in all models were: Baseline levels of cardiovascular measure (i.e., DBP, SBP or HR, respectively); age; gender; education; NYHA functional class; AH; smoking history; use of nitrates; anxiety and depressive symptoms; Type D Personality; perceived difficulty; and perceived efforts.

After controlling for possible covariates, global fatigue was found to predict diminished ΔHR (β = –0.238; *p* = 0.04) during TSST task instruction (anticipatory stress), explaining 20.5% of the variance (see Table 5). Mental fatigue was found to predict diminished ΔSBP (β = –0.244; *p* = 0.04) as well as lower ΔHR (β = –0.303; *p* = 0.01) during TSST task instruction (anticipatory stress), explaining 19.5 and 22.6% of the variance, respectively (see Table 6). Total fatigue score predicted diminished ΔSBP (β = –0.331; *p* = 0.01) and ΔHR (β = –0.324; *p* = 0.01) during TSST task instruction (anticipatory stress), explaining 21.7 and 24.0% of the variance, respectively (see Table 7). No significant associations were found between fatigue and other cardiovascular responses to TSST during other phases. There was also no association between fatigue and baseline cardiovascular measures.

**TABLE 5:** Multivariable Linear Regression model, examining global fatigue score associations with cardiovascular reactivity during Trier Social Stress Test (TSST) anticipatory stress (task instruction), while controlling for possible covariates.

Predictors	ΔHeart rate	
	β	*p*
Global fatigue score	**–0.238**	**0.039**
Baseline cardiovascular parameter	**–0.393**	**<0.001**
Gender	0.092	0.37
Age	–0.076	0.48
Education	0.070	0.50
New York Heart Association functional class	0.066	0.53
Smoking	0.065	0.55
Nitrates	0.114	0.27
Anxiety symptoms	**–0.464**	**<0.001**
Depressive symptoms	0.145	0.27
Type D personality	–0.022	0.84
Perceived efforts of the Trier Social Stress Test tasks (Visual Analog Scale)	–0.150	0.13
Perceived difficulty of the Trier Social Stress Test tasks (Visual Analog Scale)	–0.171	0.09
ANOVA and model summary		
F (df, df)	2.91 (13,128)	
*P*-value	0.002	
*R* ^2^	0.313	
*R* ^2^_Adjusted_	0.205	

**Gender (male [1]; female [2]), education (high school [1]; college/university degree [2]), NYHA functional class (I-II class [1]; III class [2]), smoking (yes [0]; no [1]), nitrates use (yes [0]; no [1]), anxiety and depressive symptom scores as measured by Hospital Anxiety and Depression Scale [HADS] (the score < 8 [0]; the score ≥ 8 [1]), Type D personality (yes [0]; no [1]); Visual analog scale range 0–5.**

*In bold significant correlations (p < 0.05).*

**TABLE 6:** Multivariable Linear Regression model, examining mental fatigue score associations with cardiovascular reactivity during Trier Social Stress Test anticipatory stress (task instruction), while controlling for possible covariates.

Predictors	ΔSystolic blood pressure		ΔHeart rate	
	β	*p*	β	*p*
Mental fatigue score	**–0.244**	**0.040**	**–0.303**	**0.011**
Baseline cardiovascular parameter	–0.141	0.144	**–0.377**	**0.001**
Gender	**0.295**	**0.005**	0.110	0.281
Age	0.071	0.491	–0.033	0.762
Education	**0.210**	**0.044**	0.103	0.321
New York Heart Association functional class	–0.044	0.674	0.019	0.852
Smoking	**0.322**	**0.003**	0.046	0.664
Nitrates	0.025	0.821	0.094	0.351
Anxiety symptoms	**–0.391**	**0.002**	**–0.412**	**0.002**
Depressive symptoms	0.244	0.061	0.140	0.270
Type D personality	0.012	0.912	–0.015	0.894
Perceived efforts of the Trier Social Stress Test tasks (Visual Analog Scale)	0.024	0.812	–0.159	0.102
Perceived difficulty of the Trier Social Stress Test tasks (Visual Analog Scale)	–0.060	0.554	**–0.222**	**0.029**
ANOVA and model summary				
F (df, df)	2.79 (13, 128)		3.16 (13,128)	
*P*-value	0.002		0.001	
*R* ^2^	0.304		0.331	
*R* ^2^_Adjusted_	0.195		0.226	

**Gender (male [1]; female [2]), education (high school [1]; college/university degree [2]), NYHA functional class (I-II class [1]; III class [2]), smoking (yes [0]; no [1]), nitrates use (yes [0]; no [1]), anxiety and depression symptom scores as measured by Hospital Anxiety and Depression Scale [HADS] (the score < 8 [0]; the score ≥ 8 [1]), Type D personality (yes [0]; no [1]); Visual analog scale range 0–5.**

*In bold significant correlations (p < 0.05).*

**TABLE 7:** Multivariable Linear Regression model, examining total fatigue score associations with cardiovascular reactivity during Trier Social Stress Test anticipatory stress (task instruction), while controlling for possible covariates.

Predictors	ΔSystolic blood Pressure		ΔHeart rate	
	β	*p*	β	*p*
Total fatigue score	**–0.315**	**0.008**	**–0.300**	**0.011**
Baseline cardiovascular parameter	–0.144	0.143	**–0.396**	**<0.001**
Gender	**0.298**	**0.006**	0.120	0.255
Age	0.073	0.485	–0.051	0.639
Education	0.176	0.096	0.083	0.424
New York Heart Association functional class	0.040	0.713	0.103	0.338
Smoking	**0.328**	**0.004**	0.086	0.437
Nitrates	0.047	0.665	0.126	0.231
Anxiety symptoms	**–0.371**	**0.003**	**–0.396**	**0.002**
Depressive symptoms	**0.296**	**0.027**	0.166	0.204
Type D personality	0.003	0.979	–0.047	0.666
Perceived efforts of the Trier Social Stress Test tasks (Visual Analog Scale)	0.027	0.784	–0.123	0.216
Perceived difficulty of the Trier Social Stress Test tasks (Visual Analog Scale)	–0.023	0.822	**–0.224**	**0.028**
ANOVA and model summary				
F (df, df)	3.12 (13, 128)		3.15 (13,128)	
*P*-value	0.002		0.001	
*R* ^2^	0.328		0.330	
*R* ^2^_Adjusted_	0.217		0.240	

**Gender (male [1]; female [2]), education (high school [1]; college/university degree [2]), NYHA functional class (I-II class [1]; III class [2]), smoking (yes [0]; no [1]), nitrates use (yes [0]; no [1]), anxiety and depression symptom scores as measured by Hospital Anxiety and Depression Scale [HADS] (the score < 8 [0]; the score ≥ 8 [1]), Type D personality (yes [0]; no [1]); Visual analog scale range 0–5.**

*In bold significant correlations (p < 0.05).*

## Discussion

The present study investigated whether subjectively experienced fatigue is associated with cardiovascular function before, during and after mental stress in CAD patients who have experienced ACS. It was hypothesized that higher subjective fatigue would be linked with diminished cardiovascular function during mental stress. In the present study, effects were observed for cardiovascular function during anticipatory stress, while participants were preparing an oral presentation as part of the TSST. Associations were found for global fatigue, mental fatigue, and total fatigue. All measures of fatigue were associated with diminished cardiovascular function during anticipatory stress. These effects remained significant even after controlling for gender, age, education, heart failure severity, history of smoking, use of nitrates, anxiety/depressive symptoms, and presence of Type D personality, as well as perceived efforts and difficulty of the task. While the observed effects for fatigue arose for anticipatory stress, fatigue was not predictive of cardiovascular function during active stress phases of the TSST.

The current study is the first to explore links between fatigue and cardiovascular responses to stress in patients with CAD. It extends prior research investigating similar associations in other populations. For example, Nolte et al. (2008) investigated associations between cardiovascular stress reactivity and naturally occurring fatigue in undergraduate women, and found that blood pressure during stress was inversely associated with fatigue. Their study drew attention to the potential role of subjective task difficulty and effort in such effects (Nolte et al., 2008; Wright et al., 2008; Wright, 2009). Taking this into consideration, in the present study, all observed associations for fatigue and cardiovascular function during anticipatory stress remained significant even while controlling for subjective difficulty and efforts, as well as for other sociodemographic and clinical parameters.

Similar to the current study results, Soetekouw et al. (1999) reported that persons with CFS exhibited suppressed HR during mental arithmetic stress compared to controls, indicating maladaptive cardiac sympathetic reactivity to mental stress. While the methodology and study population differed, the present study yielded broadly similar findings. Together, both studies further implicate diminished cardiovascular stress reactivity as an indicator of maladaptive autonomic response that characterize poor states of health (Lovallo, 2011; Hughes et al., 2018).

Despite the consistent findings with previous research (Soetekouw et al., 1999; Nolte et al., 2008), the findings of the present study contrast with other published results. For instance, Wright et al. (2008) found an association between high fatigue and higher cardiovascular reactivity to an emotional stressor in female college students. Another study by Nelesen et al. (2008) reported no effect of fatigue with regards to cardiovascular reactivity during a mental stress challenge. These different results likely arise due to their methodological and population differences, chiefly the age and disease status of the participants, but also differences in the measurement of fatigue.

The current study revealed that an apparently blunted cardiovascular response to anticipatory stress is associated with subjectively experienced fatigue in CAD patients who have experienced ACS. Phillips et al. (2013) have summarized several reasons why diminished cardiovascular stress responsivity can arise, including lower task effort, reduced stress perception, disproportionate feelings of task difficulty, reduced physiological capacity to respond, and motivational dysregulation within brain activity. In this study, even while controlling for subjective efforts and assumed difficulty of the task, associations between lower cardiovascular stress reactivity and fatigue remained, suggesting—perhaps—that the effects were related more to central dysfunction than to perceptual factors. However, it was beyond the scope of this study to investigate the reduced physiological capacity to respond to stressful stimuli or motivational dysregulation.

Neither blunted nor exaggerated stress reactions can be considered adaptive (Phillips et al., 2013). In this study it was found that in CAD patients who were more fatigued, cardiovascular reactivity was more diminished during anticipatory stress. Anticipation ordinarily helps the body to prepare for oncoming stress. When an individual is suffering with fatigue, it may lead to poor bodily preparation for the upcoming stressor, thereby compounding the maladaptive effects of fatigue. It is well established that individuals with CFS show significant changes in the function of sympathetic as well as parasympathetic nervous systems (Soetekouw et al., 1999). The sympathetic nervous system predominance in persons with chronic fatigue highlights the way in which fatigued individuals are constantly under stress (Luyten et al., 2011), which leads to sympathetic over-activity (Martínez-Martínez et al., 2014). It has been argued that constant sympathetic over-activity could be partly responsible for lost functional capacity to confront life stressors (Siegrist et al., 1989). If that is the case, the current finding of blunted anticipatory cardiovascular stress response and its independent association with fatigue in patients with CAD after ACS may reflect maladaptive cardiac functioning.

Despite the consistent findings, several limitations should be noted. First, this study was completed in a single unit of a CR clinic. Therefore, generalizability to other cohorts should not be assumed until corroborated. Secondly, the current research followed a cross-sectional study design, thus a possible causational relationship between cardiovascular stress reactivity and subjective fatigue could not be drawn and further longitudinal studies are required. Thirdly, there was no inclusion of a healthy control group, which could have allowed for comparison of the cardiovascular stress response between those with CAD after ACS, and healthy individuals otherwise.

Nevertheless, the major strength of this study is the opportunity to isolate effects of fatigue in patients with CAD, for whom subtle variations in cardiovascular function during stress is likely of greater clinical significance than in health populations. Given the preponderance of past research on healthy populations, especially college students, the present findings also serve to complement the existing literature in cardiovascular reactivity and related factors. This research also examined variety of sociodemographic and clinical characteristics, which helped to isolate possible confounders for existing associations between cardiovascular reactivity during mental stress and fatigue in study patients. Given the severe illness of the study participants, the sample size was somewhat restricted in scope, but nonetheless large enough to demonstrate important effects.

Future replications will be of high importance in larger and more diverse samples. Such research should concentrate on not only cardiovascular reactivity, but also on neuroendocrinological stress responses (i.e., cortisol responses), as such responses are intricately intertwined with physical fatigue. Fatigue is a problematic symptom commonly experienced by patients with CAD. As such, the current findings draw attention to the way diminished stress responses in fatigued patient can serve as possible indicators of adverse health that may bolster clinical understanding, as well as diagnostic evaluation and treatment.

## Conclusion

In the current study it was found that global, mental and overall fatigue were linked to cardiovascular reactivity during anticipatory stress in patients with severe cardiovascular pathology. The effects remained significant after comprehensively controlling for potential confounds, both medical, psychological, and perceptual. This is the first study to investigate psychophysiological stress response and its relationship with fatigue in an acutely ill cardiac population.

## Data Availability Statement

The original contributions presented in the study are included in the article/supplementary material, further inquiries can be directed to the corresponding author/s.

## Ethics Statement

This study involving human participants were reviewed and approved by the Kaunas Regional Ethics Committee for Biomedical Research in Lithuania. The participants provided their written informed consent to participate in this study.

## Author Contributions

JG-S: conceptualization, methodology, formal analysis, writing—original draft, review, and editing, and investigation. BH and LH: writing—review and editing. JBr and NM: conceptualization and supervision. AB, JBu, JN, NK, and NM: revision and supervision. All authors provided critical revision to its further development, read and approved the final manuscript.

## Conflict of Interest

JG-S served as a consultant at FACITtrans. In the past several years JBu has been serving as a consultant to Cogstate, Ltd. The remaining authors declare that the research was conducted in the absence of any commercial or financial relationships that could be construed as a potential conflict of interest.

## Publisher’s Note

All claims expressed in this article are solely those of the authors and do not necessarily represent those of their affiliated organizations, or those of the publisher, the editors and the reviewers. Any product that may be evaluated in this article, or claim that may be made by its manufacturer, is not guaranteed or endorsed by the publisher.
